# Analysis of Melatonin-Modulating Effects Against Tartrazine-Induced Neurotoxicity in Male Rats: Biochemical, Pathological and Immunohistochemical Markers

**DOI:** 10.1007/s11064-022-03723-9

**Published:** 2022-08-26

**Authors:** Amina E. Essawy, Ahmed Ibrahiem Mohamed, Rania Gaber Ali, Awatef M. Ali, Heba Mohamed Abdou

**Affiliations:** 1 Department of Zoology, Faculty of Science, Alexandria University, Alexandria, Egypt; 2 Department of Pathology, Faculty of Medicine, Alexandria University, Alexandria, Egypt

**Keywords:** Tartrazine, Melatonin, Oxidative stress, Neurotransmitters, Cytokines, Immunohistochemistry

## Abstract

Tartrazine (E-102) is one of the most widely used artificial food azo-colors that can be metabolized to highly sensitizing aromatic amines such as sulphanilic acid. These metabolites are oxidized to N-hydroxy derivatives that cause neurotoxicity. Melatonin is a neurohormone. That possesses a free-radical scavenging effect. The present work was mainly designed to evaluate the possible ameliorative role of melatonin against tartrazine induced neurotoxicity in cerebral cortex and cerebellum of male rats. Adult male rats were administered orally with tartrazine (7.5 mg/kg) with or without melatonin (10 mg/kg) daily for four weeks. The data revealed that tartrazine induced redox disruptions as measured by significant (p < 0.05) increased malondialdehyde (MDA) level and inhibition of (GSH) concentration and catalase (CAT), superoxide dismutase (SOD) and glutathione peroxidase (GPx) antioxidant enzyme activities. Besides, brain acetyl cholin (Ach) and gamma-aminobutyric acid (GABA) were elevated while, dopamine (DA) was depleted in trtrazine -treated rats. Moreover, tartrazine caused a significant (p < 0.05) increase in the brain interleukin-6 (IL-6), interleukin-1β (IL-1 β) and tumor necrosis factor-α (TNFα). At the tissue level, tartrazine caused severe histopathological changes in the cerebellum and cerebral cortex of rats. The immunohistochemical results elucidated strong positive expression for Caspase-3 and GFAP and weak immune reaction for BcL2 and synaptophysin in tatrazine- treated rats. The administration of melatonin to tartrazine -administered rats remarkably alleviated all the aforementioned tartrzine-induced effects. It could be concluded that, melatonin has a potent ameliorative effect against tartrazine induced neurotoxicity via the attenuation of oxidative/antioxidative responses.

**Supplementary Information:**

The online version contains supplementary material available at 10.1007/s11064-022-03723-9.

## Introduction

Tartrazine (E-102) is one of the most widely used artificial food azo-colors extracted from coal tar [1]. It contains an azo group (N = N), which is very harmful to living organisms [2]. The azo compounds, with the N = N functional groups and aromatic rings linked to them, are reductively cleaved into aromatic amines (as sulfanilic acid) via the gut microbiota [3]. Some of these amines are toxic, mutagenic and carcinogenic [4]. These metabolites of tartrazine can generate reactive oxygen species (ROS), induce oxidative stress, and affect the architectures of different organs and their biochemical profiles such as liver, kidney, stomach and pancreas [5–7]. Demirkol et al. [8] reported that, when tartrazine is reduced totally into aromatic amines, they are oxidized to N-hydroxy derivatives by the enzymatic system of cytochrome P450. These derivatives can cross the blood brain barrier and generate reactive oxygen species in brain. Saxena and Sharma [9] stated that, ingestion of tartrazine is concerned with plentiful health issue for instance anxiety, headaches, blurry vision, and sleep disturbance. The most detrimental effects of tartrazine have been related to deoxyribonucleic acid (DNA) destruction [10].

There is a recent trend to use antioxidants to neutralize the suspected neurotoxic effects of food additives. López-Armas et al., [11] reported that, the chemical properties and the antioxidant activity of melatonin provide significant neuroprotection in neurodegenerative disease and neuroinflammation.

Melatonin (5-methoxy-N-acetyltryptamine) is a neurohormone produced by pineal gland in response to signals received from the postganglionic fibers [12].

Production of melatonin in the body is governed by the circadian rhythm and it functions as (1) anti-apoptotic via the inhibition of apoptotic cascade; (2) antioxidant via scavenging reactive oxidative species, harmful to macromolecules and triggering apoptosis; (3) activation of mitochondrial metabolism; and (4) anti-inflammatory by either inhibition microglia activation or blockage of enzymes for example cyclooxygenase2 and inducible nitric oxide synthase which synthesize proinflammatory mediators and nitric oxide, respectively [13]. Extensive research has certified the antioxidant action of melatonin and its beneficial potential in preserving the nervous tissue from oxidative damage [14, 15].

Additionally, there are no stated side effects concomitant with long-term application of melatonin at both pharmacological and physiological doses [16].

The present study aimed to assess the in vivo effect of oxidative stress induced by tartrazine and the neuroprotective role of melatonin on the injured brain. Herein, we have investigated the effect of melatonin aginst tartrazine -induced alterations in oxidative-antioxidative status, Ach, GABA and DA neurotransmitters, cytokines and cerebral and cerebellar cortex histopathology and immunohistochemistry.

## Materials and Methods

### Chemical and Reagents

All chemicals and reagents used were of high analytical grade. Tartrazine ( C16H9N4Na30O9S2, ≥ 95% purity) was purchased from Sigma-Aldrich (St Louis, MO, USA), melatonin capsules were obtained from Puritan’s Pride, INC (USA). Kits were purchased from Biodiagnostic and Research Reagents Co. (Cairo, Egypt). The Enzyme-linked immunosorbent assay ElISA kits were purchased from Sigma Chemical Co. (Sigma-Aldrich Chemie GmbH Germany).

### Experimental Design

The present study was carried out on 40 male Wistar albino rats (12 weeks old, weight 180–200 g), obtained from Faculty of Agriculture, Alexandria University, Egypt. They were housed in stainless steel wire cages, placed in a well-ventilated laboratory (temperature: 22 ± 2 °C) and kept on basal diet and tap water ad libitum. All experimental procedures and animal handling were confirmed to the guidelines approved by Alexandria University Institutional Animal Care and Use Committee (ALEXU- IACUC), (Approval number: AU 04 19 01 26 1 01).

Animals were randomly divided into four equal groups of 10 rats each, and treated as follow:

Group 1 (control): The rats were orally administered 0.5 ml distilled water (vehicle) daily for 4 weeks.

Group 2 (melatonine): The rats were orally administered melatonin at a dose of 10 mg/kg dissolved in 0.5 ml dist. water [17].

Group 3 (tartrazine): The rats were administered tartrazine orally at a dose of 7.5 mg/kg/day dissolved in 0.5 ml dist water [6].

Group 4 (tartrazine + melatonin): The rats were orally administered tartrazine at a dose of 7.5 mg/kg/day and in combination with melatonin at dose of 10 mg/kg/day. The experiment extended for 4 weeks.

#### Biochemical analysis

After 4 weeks, the rats were fasted overnight and sacrificed under anesthesia. Cerebral cortex of six rats from each group was quickly removed, washed with cold normal saline, minced and homogenized (10% w/v) in 4 ml ice-cold sucrose buffer (0.25 M). The homogenate was centrifuged at 10,000 xg for 20 min at4 °C. The supernatant was stored at − 80 °C until use in the assessment of biochemical parameters.

### Determination of Oxidative Stress Markers

Malondialdhyde (MDA), the end product of Lipid peroxidation (LPO) was determined by the method [18]. Level of reduced glutathione (GSH) was measured by the method of [19]. The activities of the enzymatic antioxidants, superoxide dismutase (SOD), Glutathione peroxidase (GPX) and catalase (CAT) were evaluated by the method of Misra and Fridovich [20], Chiu et al. [21] and Aebi [22], respectively.

### Estimation of Neurotransmitters

Acetylcholin (ACh), Dopamine (DA) and gamma-aminobutyric acid (GABA) were measured by the relevant ELISA kit as directed by the manufacturer’s test recommendations ( Ach, CAT No.: CEA912Ge; Cloud-Clone Corp.(CCC) USA; DA, CAT No.: CEA851Ge, Cloud-Clone Corp.(CCC) USA; GABA, CAT No.: CEA900Ge, Cloud-Clone Corp.(CCC) USA).

### Determination of Inflammatory Markers

Quantitative detection of brain interleukin-6 (IL-6) and interleukin-1β (IL-1 β) were detected by ELISA kits according to the method of [23], while tumor necrosis factor–alpha (TNF-α) was estimated by ELISA kit according to Hedayati et al. [24].

### Histological and Immunohistochemical Analysis

The cerebral cortex and cerebellum of four animals from each experimental group were dissected out after formalin perfusion. Dehydration, clearing and paraffin embedding were followed according to [25]. Sections were stained with haematoxylin/eosin and examined under a light microscope.

For immunehistochemical staining, successive 5 μm thick cerebral cortex paraffin sections were loaded on positively charged slides and incubated overnight at 37 _C, to assess the expression of caspase-3, Bcl-2 according to technique described by Hsu et al. [26] and GFAP according to [27]. Primary antibodies of caspase-3 (ab4051, abcam, Inc), Bcl2 (ab196495, abcam, Inc) and GFAP (Ab-1,Clone GA-5, Lab Vision Corporation, Medico Co., Egypt) were applied for immuno-localisation according to the manufacturer's protocol. For immunostaining of synaptophysin, deparaffinized sections of cerebellar cortex were incubated with rabbit polyclonal primary antibody; Synaptophysin antibody(PA5-27,286, Thermo Scientific, USA). Binding of primary antibody was demonstrated using Thermo Scientific Super Picture™ Polymer Detection Kit.

### Statistical Analysis

All data are expressed as means ± standard error (SE) of number of experimental animals (n = 6). The statistical significance level of treatment effects was evaluated by one way analysis of variance using SPSS version 22 (SPSS, IBM, USA) and post hoc comparison between groups were obtained by Duncan’s Multiple Range Test (Dmrt). Values were considered statistically significant when p < 0.051 [28].

## Results

### Effect of Melatonin on Tartrazin-Induced Oxidative Impairments

**Fig. 1:**
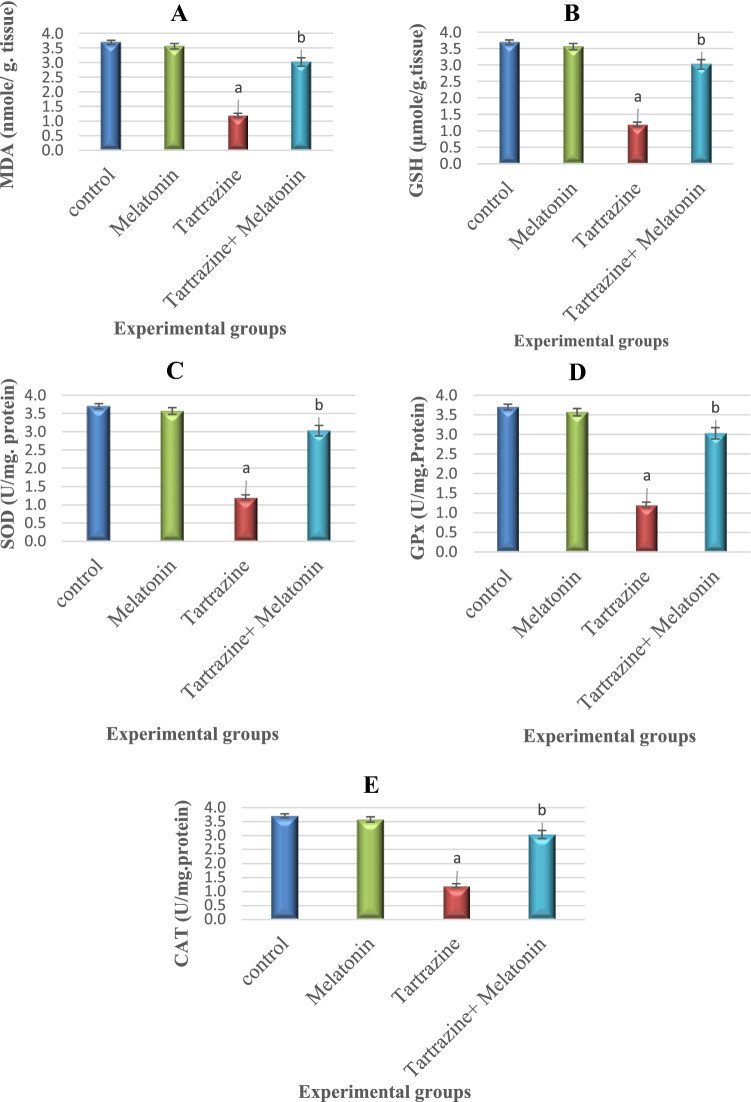
Effect of tartrazine, melatonine and their combination on the level of **A**: MDA, **B**: GSH, **C**: SOD, **D**: GPX and **E**: CAT in the cerebral cortex of rats. All the data were analyzed using one-way ANOVA followed by LSD post Hoc test. Values are expressed as mean ± SD.; n = 6 rats for each group. a The mean values are significantly different compared to the control group at p < 0.05. b The mean values are significantly different compared to the tartrazin group at p < 0.05. For MDA: Variation between groups = 3328.669, Variation within groups = 15.846 So, F = 210.065. For GSH: Variation between groups = 19.764, Variation within groups = .519 So, F = 38.098. For SOD: Variation between groups = 27.813, Variation within groups = .124 So, F = 224.298. For GPx: Variation between groups = 8.333, Variation within groups = .009 So, F = 925.889. For CAT: Variation between groups = 7.987, Variation within groups = .010 So, F = 798.7

The oxidative status of the cerebral cortex was evaluated by monitoring the perturbations in the antioxidant parameters; CAT, SOD, and GPx, as well as the levels of GSH and MDA (Fig. 1). Administration of melatonin alone induced an insignificant (p > 0.05) changes in the activities of the antioxidant enzyme and the level of MDA, while it caused a significant (p < 0.05) elevation of GSH level compared to the control group. Rats administered with tartrazine showed a significant diminution in the activities of CAT, SOD, GPX and GSH with notably increased brain MDA level compared to control rats. Co-administration of melatonin with tartrazine, significantly reverted back the level of MDA and GSH and the activities of CAT, SOD, GPx as compared to tartrazine-intoxicated group.

### Melatonin Modulates Tartrazine-Induced Changes in Neurotransmitters Levels

**Fig. 2:**
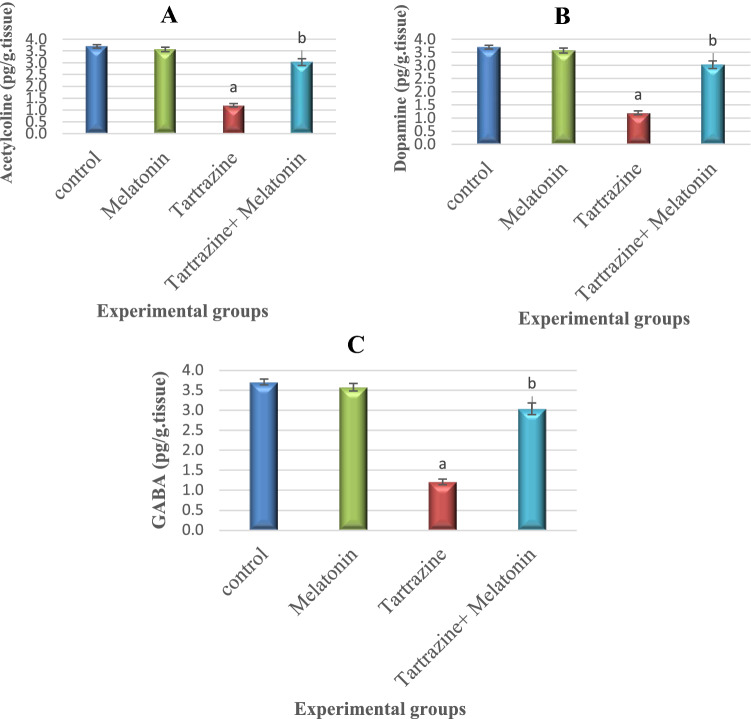
Effect of tartrazine, melatonine and their combination on the level of **A**: acetylcholine (Ach), **B**: DA and **C**: GABA in the cerebral cortex of rats. All the data were analyzed using one-way ANOVA followed by LSD post Hoc test. Values are expressed as mean ± SD.; n = 6 rats for each group. a The mean values are significantly different compared to the control group at p < 0.05. b The mean values are significantly different compared to the tartrazin group at p < 0.05. For Ach: Variation between groups = 1493.395, Variation within groups = 1.149So, F = 1299.735. For DA: Variation between groups = 1242.807, Variation within groups = .824 So, F = 1508.261. For GABA: Variation between groups = 503.687, Variation within groups = 3.945 So, F = 127.677

Further, we found that melatonin supplementation haltered the increase in Ach and GABA as well as the decrease in DA in the tartrazine-intoxicated rats. Figure 2 illustrates the effect of tartrazine and/or melatonin on brain Ach, GABA and DA. Tartrazine administration resulted in a significant (p < 0.05) high level of Ach and GABA and a significant low level of DA than the control group. However, co-administration of both melatonin and tartrazine attenuated the levels of Ach, GABA and DA in rat’s brain, compared with that of the tartrazine-treated group. The sole treatment of melatonin was safe and showed an insignificant (p > 0.05) change in the neurotransmitters levels when compared to the control.

### Melatonin Attenuates Tartrazine-Induced Neuroinflammation

**Fig. 3:**
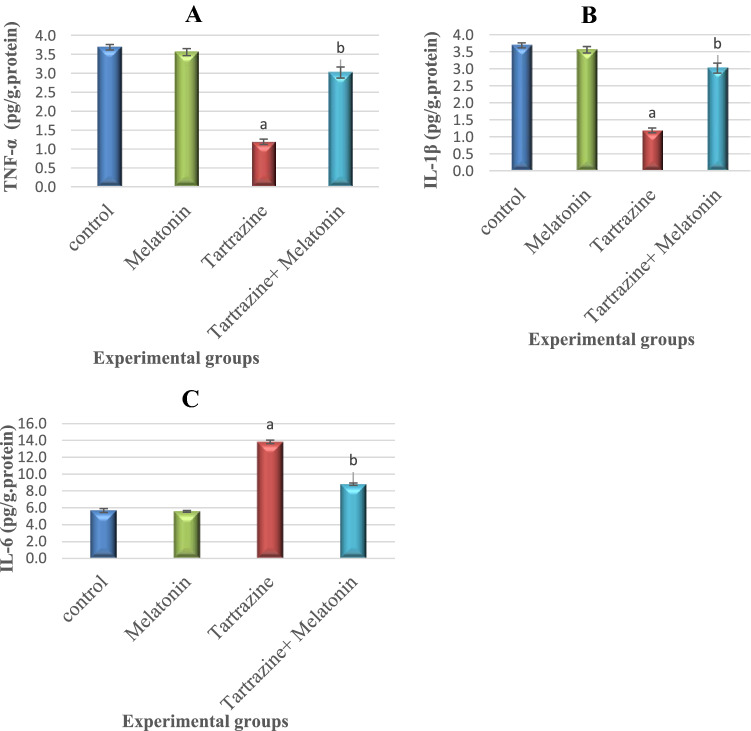
Effect of tartrazine, melatonine and their combination on the level of A: tumor necrosis factor–alpha (TNF-α), B: interleukin-1β (IL-1 β) and C: interleukin-6 (IL-6) in the cerebral cortex of rats. All the data were analyzed using one-way ANOVA followed by LSD post Hoc test. Values are expressed as mean ± SD.; n = 6 rats for each group. a The mean values are significantly different compared to the control group at p < 0.05. b The mean values are significantly different compared to the tartrazin group at p < 0.05. For TNF-α: Variation between groups = 107.773, Variation within groups = .047 So, F = 2293.043. For IL-1 β: Variation between groups = 517.508, Variation within groups = 3.386 So, F = 152.838. For IL-6: Variation between groups = 90.251, Variation within groups = .031So, F = 2911.32

As shown in Fig. 3, tartrazine administration induced significant (P < 0.05) elevation.

in TNF-α, IL-1β and IL-6 levels compared to control and melatonin- treated groups.

Co-administration of melatonin with tartrazine elicited significant (P < 0.05) reduction in the levels of these proinfalmmatory markers as compared to tartrazine group.

### Histological Observations

**Fig. 4:**
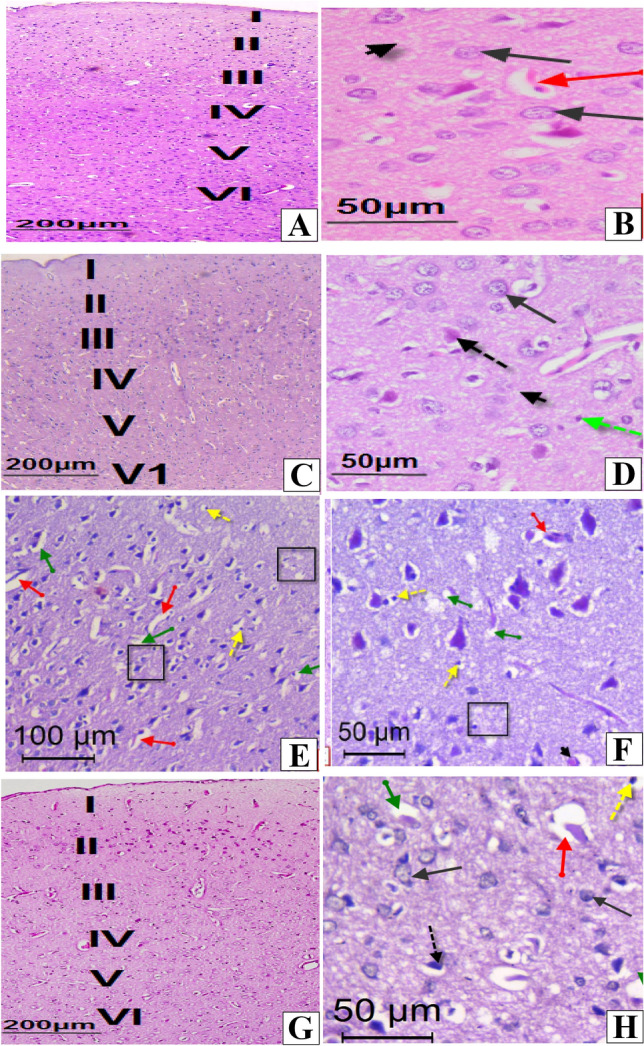
Photomicrographs of sections in the cerebral cortex of rats. **A**&**B:** Sections in control rats, showing normal histological structure of the cerebral cortex layers; molecular layer (1), outer granular layer (II),outer pyramidal layer (III), inner granular layer (IV), inner pyramidal layer(V) and the multiform layer (VI). Note, normal granular cells (black arrows), blood vessel (red arrow) and normal neuropil (black arrow’s head). **C**&**D**: Sections in melatonin-treated rat showing normal structure of the cerebral cortex with its 6 cellular layers (I–VI). Note, normal pyramidal cells (black dotted arrow), granule cells (black arrow), neuroglial cells (green dotted arrow), blood vessel (red arrow) and normal neuropil (black arrow’s head). **E**&**F**: Sections in tartrazin-treated rats, showing degenerated pyramidal neurons (black Square), irregular pyramidal cells with darkly stained nuclei (blue dotted arrows), pericellular vacuoles (green arrow), vacuolated neuropil (red arrows head), pyknotic nuclei (yellow dotted arrows), red neuron (black arrows head), pericellular edema (green arrows), dilated and congested blood vessels (red arrows) and glail cells (black circle). **G**&**H**: Sections in, showing improvement in the granular cells (black arrows) and pyramidal cells (black dotted arrow). Some dilated blood vessels (red arrow), few neurons with pyknotic nuclei (yellow dotted arrow) and few pericellular vacuoles (green arrow) were observed. (**H**&**E** stain)

In control and melatonin supplemented rats (Figs. 4A–D), the sections of the cerebral cortex showed the normal characteristic structure of the cerebral cortex and its layers; molecular, outer granular, outer pyramidalr, inner granular, inner pyramidal and multiform layers.

However, in tatrazine- treated rats, the outer pyramidal layer showed large irregular pyramidal cells with darkly stained nuclei and pericellular edema, vacuolated neuropil with dark nucleated glial cells, red cells and dilated and congested blood vessels (Figs. 4E and F). In tatrazine -treated rats that supplemented with melatonin (Figs. 4G and H), the cerebral cortex layers displayed obvious recovery in their histological pattern in spite of little pyknotic nuclei and some vacuoles still found.

**Fig. 5:**
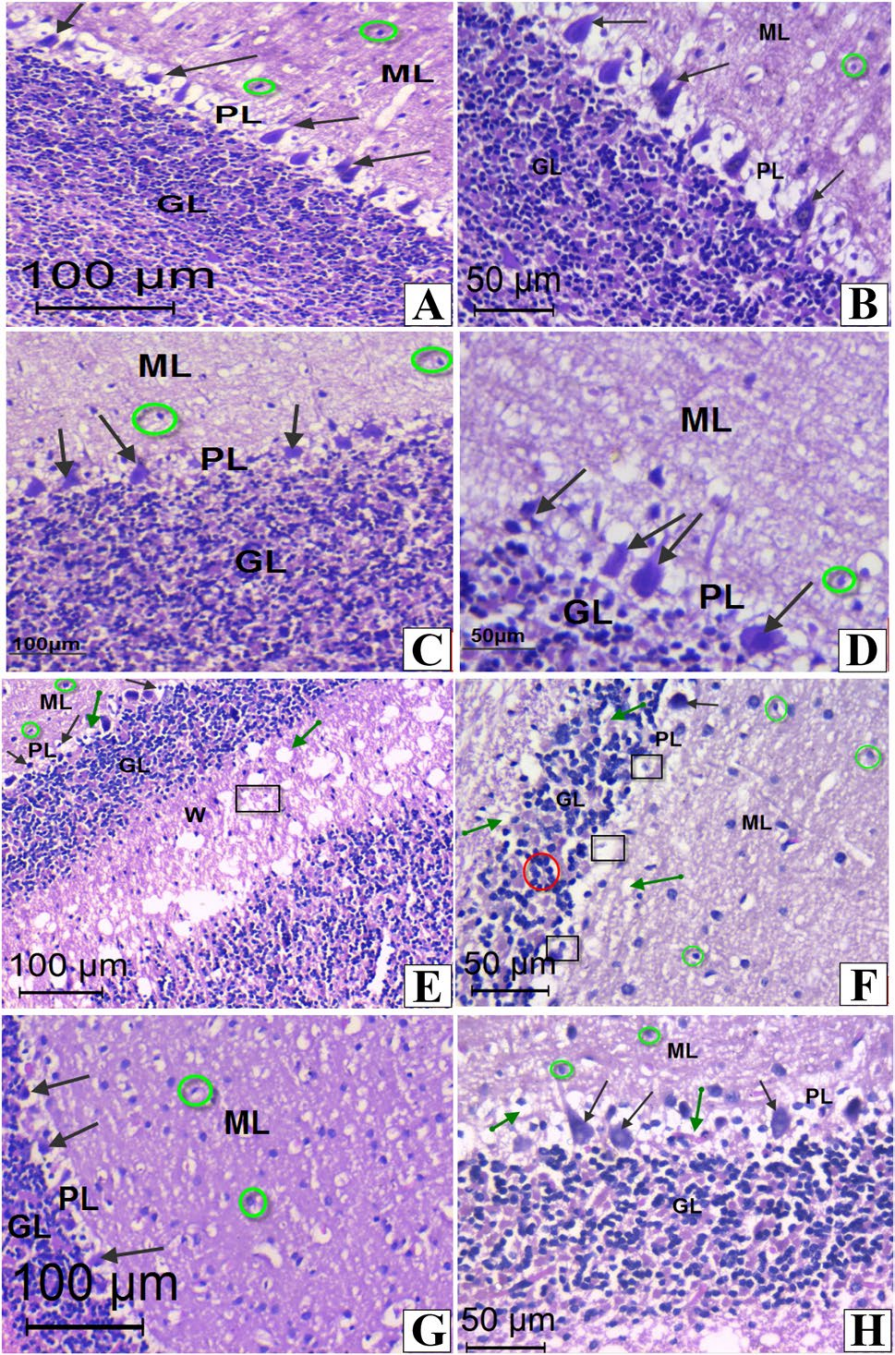
Photomicrographs of sections in the cerebellar cortex of rats. **A**&**B:** Sections in control rats, showing the normal three layers; outer molecular (ML) with interneurons (green circle), purkinje layer (PL) with purkinje cells (black arrows) and inner granular cell layer (GL) consisting of small rounded granular cells. **C**&**D**: Sections in melatonin-treated rats showing normal structure of the previously mentioned three cellular layers, outer molecular, purkinje layer and inner granular cell layer. **E**&**F:** Sections in the cerebellar cortex from tartrazin-treated rat, showing molecular layer with prominent perineuronal vacuoles, irregular shaped Purkinje cells with darkly stained nuclei (black arrows) and remnants of degenerated Purkinje cells (black squares), whit matter (W) with vacuoles (green arrows), granular layer (GL) consisting of clumped granular cells with deeply stained nuclei (red circle), areas of degenerated cells and fibers (black square) and scattered interneuron cells (green circles). **G**&**H** sections from tartarazin + melatonin-treated rat, showing improvement in the structure of molecular layer with interneuron cells (green circle), purkinje cells (black arrows) in purkinje layer (PL) and granular cells in granular layer (GL). Few vacuolated cells ( green arrows) were noticed

The cerebellar cortex of the control and melatonin -treated rats showed the normal histological features demarcated cortical cell layers the molecular, Purkinje cell and granular layers (Figs. 5A–D). Conversely, cerebellar sections from tartrazine-intoxicated rats exhibited severe and widespread fragmentation of granule cell layer with neuronal loss (Figs. 5E and F). Moreover, Purkinje cells had shrunken pyknotic nuclei and widened pericellular empty spaces. Interestingly, the treatment of rats with melatonin diminished tatrazine -induced cerebellar lesions and the cerebellar layers were generally preserved (Figs. 5G and H).

### Immunohistochemical Observations

**Fig. 6:**
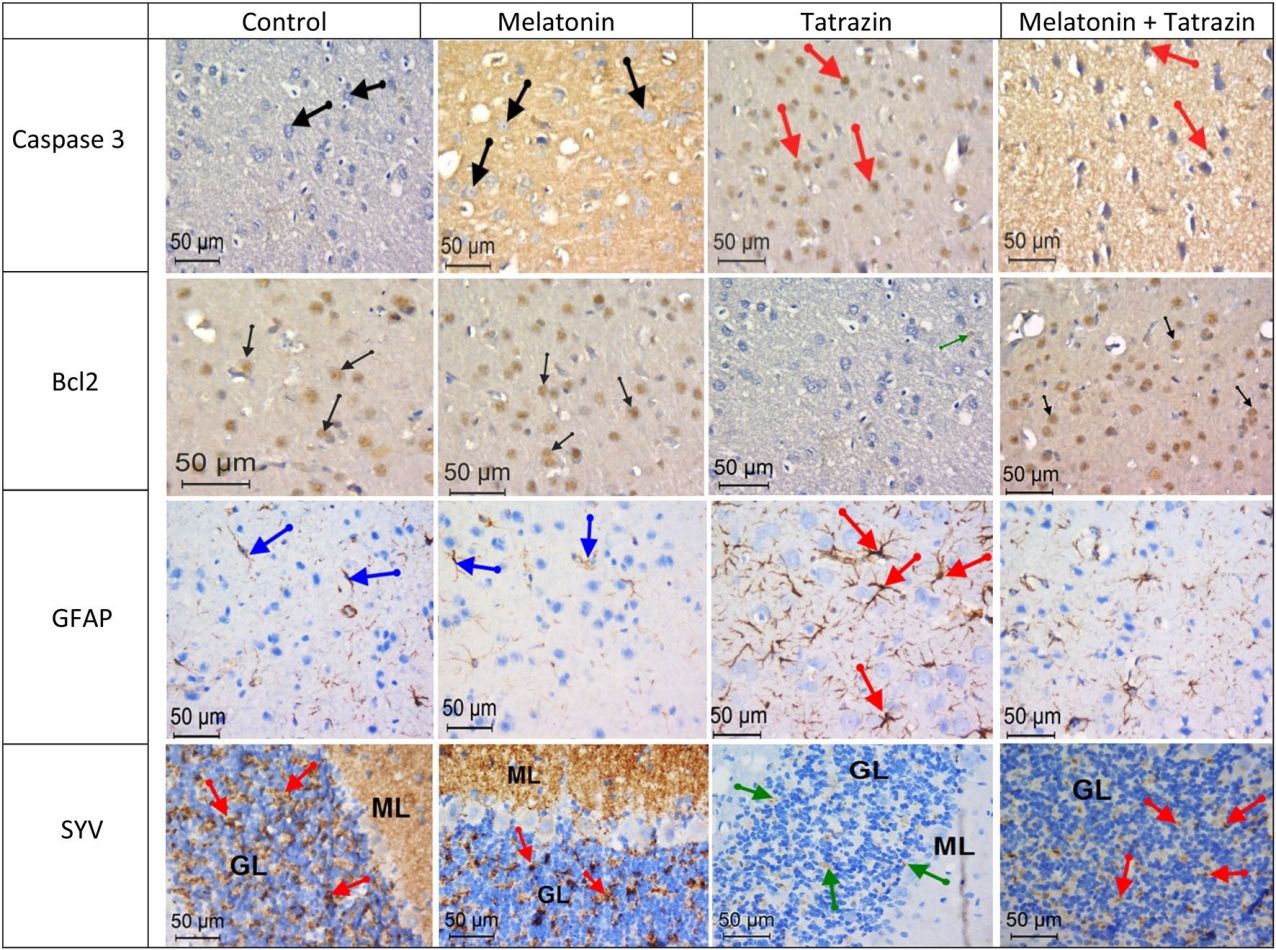
Photomicrographs of coronal sections in rats brain, showing immunoreactivity for Caspase-3, Bcl2, GFAP ( in cerebral cortex) and CYV( in cerebllar cortex). Sections in control and melatonin treated rats showed negative or faint positive reaction for both Caspase-3 and GFAP and a strong stain affinity for Bcl2 and CYV. On the other hand, brain sections from tartrazine-treated rats showed strong reaction for Caspase-3 and GFAP and weak reaction for Bcl2 and CYV. Cortex coronal sections in rats co-treated with melatonin + tartrazine showed decreased number of both caspase-3 and GFAP positive cells and increased number of both Bcl2 and CYV positive neurons

Figure 6 showed weak immune-reactivity for caspase-3 and GFAP in cerebral cortex of control and melatonin-treated rats. However, the cerebral cortex of tartrazine-treated animals showed intensive positive immune expression for the two antigens. Rats treated with tartrazine in combination with melatonin showed weak immune-staining for caspase-3 and GFAP in comparison to tartrazine-intoxicated group.

Moreover, strong immunoreactivity for B-cl2 was observed in cerebral cortex of control and melatonin-treated animals. However, weak B-cl2 immune-reactivity was recorded in tartrazine -treated rats. Administration of tartrazin + melatonin improved the immunostaining of neurons for B-cl 2.

In control and melatonin -treated rats, the SYP immunoreactivity was strongly or moderately detected in the cerebellar tissue, while in tatrazine—treated rats such activity was markedly decreased. On the other side, in tatrazine treated rats pre supplemented with melatonin a remarkable restore in the SYP immunoreactivity was noticed that more or less similar to controls.

## Discussion

Tartrazine is one of the most used food additives all over the world. Its metabolites accumulate in the brain and cause prolonged neurotoxicity, leading to neurodegeneration.

On the other hand, melatonin is a neurohormone that has a wide-ranging neuroprotective role. It is a promising potential therapeutic neuroprotective agent for some neurological disorders.

The present study manifested that tartrazine increased oxidative stress in the rats, since it induced an elevation in MDA level associated with reduction in GSH level in the brain of the treated rats. These results are consistent with the findings of Mahmoud et al. [29] who reported that, administration of tartrazine caused marked reduction in GSH level and remarkable elevation in MDA, a byproduct of lipid peroxidation. When azo- reductases and peroxidases catalyze azo-food dyes, semiquinone radicals and aromatic amines are generated and led to the production of superoxide radicals, hydroxyl radicals, and hydrogen peroxide [8]. Brain tissue contains large amounts of polyunsaturated fatty acids, which are predominantly vulnerable to free radical damage [30].

A significant improvement in the level of the oxidative stress markers (MDA and GSH) was recorded in the brain of rats treated with melatonin + tartrazine, indicating the antioxidant potential of melatonin against neurotoxicity. These results are in accordance with findings of Sadek et al. [17] who studied the signaling pathways and related mechanisms underlying melatonin-induced neuroprotection in AlCl_3_-initiated rat’s neurotoxicity. Melatonin provokes intracellular GSH content by effecting on gamma-glutamyl cysteine synthetase [31]. It was an emphasis on the influence of melatonin on mitochondria, where it safeguards respiratory electron flux, diminishes oxidant generation, lessens electron leakage and mitigates the mitochondrial transition pore permeability [32].

Moreover, administration of tartrazine induced depletion in the activity of SOD, GPX and CAT in the brain of rats. Similarly, [33] studied the toxic impact of tartrazine on deactivating endogenous antioxidant defense enzymes in rat brain tissue. Intestinal microbiot metabolize tartrazine into aromatic amines, which form ROS as a part of their metabolic products [33]. As a consequence to ROS generation, SOD commenced to be used up to preclude the cell death by these toxic radicals [34]. The diminished activity of CAT in brain tissue was by virtue of excess adequateness of hydrogen peroxide in the biological systems which, in turn, yields peroxyl and hydroxyl radicals. Besides, brain tissue composed of enormous amounts of polyunsaturated fatty acids, which are principally exposed to free radical insult [35].

Co-administration of melatonin with tartrazine restores the activities of SOD, GPX and CAT in a rat’s brain. These results are concomitant with Metwally et al. [36] who stated that melatonin supplementation remarkably ameliorates the activities of SOD, CAT and GPx in the kidneys, brain and liver of the diabetic rats. El-Beltagy et al. [37] mentioned that melatonin up-regulates antioxidant enzymes (SOD, CAT and GPX). As melatonin can easily penetrate the blood–brain barrier [38], trigger brain resilience against neurodegenerative diseases and enhance neuronal survival and neuroplasticity [36].

Reiter et al. [39], reported that,the experimental evidence supports melatonin’s actions as a direct free radical, as an indirect antioxidant when stimulating antioxidant enzymes, its stimulation of the synthesis of glutathione, its ability to augment the activities of other antioxidants(or vice versa), its protection of antioxidative enzymes from oxidative damage, and its ability to increase the efficiency of mitochondrial electron transport chain thereby lowering electron leakage and reducing free radical generation..

The present study indicated that the levels of Ach and GABA in the brain were increased after tartrazine -treatment, but it promoted decline in the level of DA. These results are consistent with those of Eman et al. [40] who observed that ingestion of tartrazine led to elevation in GABA levels in the brain, but it contributed to the diminution in the level of DA. Free radicals may deactivate the voltage-dependent calcium channels which in turn elevate the GABA level [40]. Ablat et al. [41] reported that, the significant loss of DA in the striatum plays a critical role in the pathogenesis of PD, which can result in low DA and high Ach levels. Furthermore, free radicals could deteriorate AChE biosynthesis [42]. Co-administration of tartrazine + melatonin significantly decreased the levels of Ach and GABA, while significantly increased the level of DA in rat’s brain. These results are in accordance with those of [31, 43] who reported that melatonin application clearly increases AChE activities in brain, suggesting that melatonin may orchestrate cholinergic functioning. This confirmed the potent protective effects of melatonin with regard to revamp cholinergic neurotransmission. Besides, melatonin may suppress enzymatic degradation of DA which incites the elevation of this monoamine.

Consistently, our results showed elevation in the levels of TNF-α, IL-1β and IL-6 in the brain of tartrazine -treated rats. These findings are going hand to hand with Demirkol et al. [8] who observed upregulation in IL-1β and IL-6 after long-term tartrazine exposure.

Co-administration of melatonin with tartrazine elicits reduction in the levels of TNF-α, IL-1β and IL-6 in rat’s brain. Our observations are consistent with the findings of Yang et al. [44] who indicated that melatonin has a powerful effect to mitigate neuro-inflammatory action caused by intestinal ischemia/reperfusion. They demonstrated that melatonin exerts anti-apoptic and antioxidative actions; hence, melatonin may safeguard the neural tissue from secondary injury. Additionally, the neuroprotective influence of melatonin by mitigating microgliosis astroglosis besides to downregulation of the expression of pro-inflammatory cytokines [45].

Our histopathological investigations showed that administration of tartrazine induced neuronal loss, vacuolar degeneration, and several other histopathological alterations in the cellular layers of both cerebellar—and cerebral cortex. These findings go parallel with the study of [29, 46] who found that tartrazine can induce neuro-degenerative changes, chromatolysis, pyknosis and apoptotic cell death in rat brain. These alterations could be attributed to the aromatic amines that enhance ROS production.

Supplementation of melatonin in combination with tartrazine showed marked improvement in the histological appearance of the cerebellar—and cerebral cortex neurons by suppressing ROS.

mediated reactions [32]. Pandi-Perumal et al*.* [47] reported that, melatonin has been shown to arrest neuropathological changes not only by scavenging free radicals but also by increasing the antioxidant enzymes and by attenuating free radicals formation via astrocytes and microglial cells.

The immuno-histochemical results of the present work revealed a strong positive expression for caspse-3 and GFAP and weak expression for Bcl-2 in cerebral cortex of tartrazine -treated rats. On the other hand, examination of cerebellar cortex of this group of rats showed a weak expression for synaptophysin (SYN) protein.

Caspase3 acts as a key effector molecule regulating the process of apoptosis [48]. Abd-Elhakim et al. [49] observed increased caspase-3 expression in rats exposed to tartrazine and the natural colorant cholorophyll. This could be due to oxidative stress that activates ROS-mitochondria-Casp3-apoptosis cascades.

GFAP is an intermediate filament protein found in astrocytes during brain injury. Strong GFAP expression was recorded in the brain of the tartrazine-treated rats which could be attributed to astrocytes activation [46]. Previous studies demonstrated that neurotoxins can induce astrocytes proliferation with increased production of GFAP [50, 51].

Bcl-2 fulfills an anti-apoptotic roll, induces cell cycling and strengths cell resistance to apoptosis [52]. [53] indicated that administration of azo dyes, Sunset Yellow or Allura Red modifies the expression level of the apoptosis regulatory protein Bcl-2.

Synaptophysin (SYN) is an integral membrane protein of synaptic vesicles which maintains synaptic transmission and fulfilling an essential regulatory role in synaptogenesis [54]. Strong or moderate SYN immune-reactivity is usually associated with regular neurotransmission of nerve impulses [55]. Conversely, weak SYN reactivity inferred to impaired synaptic function and abnormal axon transport and conducing to cognitive impairments [56].

In the present study, the decreased expression of SYN in ROT- treated rats could be due to a possible inhibition of SYN protein biosynthesis through DNA fragmentation. Tartrazine has been shown to induce DNA fragmentation in leucocytes of rats as detected by comet assay [6]. On the other hand, positive immunostaining for SYN was recorded in rats treated with melatonin + tartrazine. These results are in accordance with those of El-Beltagy et al*.* [37] who showed the ameliorative action of melatonin against deltamethrin induced weak immune-reactivity of SYN in the brain of pregnant rats and their offspring.

The neuroprotective impact of melatonin on brain is due to its unique interactions with specific receptors contributing to maintenance of neuronal cells [57]. Additionally, melatonin can maintain cellular homeostasis and survival by modulating apoptosis and inflammation following several types of brain injury [58].

## Conclusions

The outcomes of our studies are of clinical importance, particularly the use of melatonin as safe neuroprotective agent against tartrazine neurotoxicity. The potential useful effects of melatonin could be via inhibition of free-radical production, scavenging of ROS, and reactivation of antioxidant defense systems. These properties qualify melatonin to be a promising potential therapeutic neuroprotective agent, with no side effects, for brain injury.

## Supplementary Information

Below is the link to the electronic supplementary material.

Supplementary file1 (DOCX 18 kb)

Supplementary file2 (DOCX 16 kb)

## Data Availability

All the data and material were available. The data of this article are included within the article and its additional files.
