# Supplementary material for: Analysis of Melatonin-Modulating Effects Against Tartrazine-Induced Neurotoxicity in Male Rats: Biochemical, Pathological and Immunohistochemical Markers
Source: Neurochem Res. 2022 Aug 26;48(1):131–41. doi: 10.1007/s11064-022-03723-9 (PMC9823072; doi:10.1007/s11064-022-03723-9)
Supplement: Supplementary file 1 — Supplementary file1 (DOCX 18 kb) [file 11064_2022_3723_MOESM1_ESM.docx]

Figures Captions

**Fig. 1:** Effect of tartrazine , melatonine and their combination on the level of A:malondialdehyde (MDA), B: reduced glutathione (GSH), C: superoxide dismutase (SOD), D: glutathione peroxidase (GPX) and E: catalase (CAT) in the cerebral cortex of rats. All the data were analyzed using one-way ANOVA followed by LSD post Hoc test. Values are expressed as mean ± SD.; n = 6 rats for each group. a The mean values are significantly different compared to the control group at p< 0.05. b The mean values are significantly different compared to the tartrazin group at p< 0.05.

For MDA: Variation between groups = 3328.669, Variation within groups = 15.846 So, F = 210.065

For GSH: Variation between groups = 19.764, Variation within groups= .519 So, F = 38.098

For SOD: Variation between groups = 27.813, Variation within groups= .124 So, F = 224.298

For GPx: Variation between groups = 8.333, Variation within groups= .009 So, F = 925.889

For CAT: Variation between groups = 7.987, Variation within groups= .010 So, F = 798.7

**Fig. 2:** Effect of tartrazine, melatonine and their combination on the level of A: acetylcholine (Ach), B: dopamine (DA) and C: gamma-aminobutyric acid (GABA) in the cerebral cortex of rats. All the data were analyzed using one-way ANOVA followed by LSD post Hoc test. Values are expressed as mean ± SD.; n = 6 rats for each group. a The mean values are significantly different compared to the control group at p< 0.05. b The mean values are significantly different compared to the tartrazin group at p< 0.05.

For Ach: Variation between groups = 1493.395, Variation within groups= 1.149So, F = 1299.735

For DA: Variation between groups = 1242.807, Variation within groups= .824 So, F = 1508.261

For GABA: Variation between groups = 503.687, Variation within groups= 3.945 So, F = 127.677

**Fig. 3:** Effect of tartrazine , melatonine and their combination on the level of A: tumor necrosis factor–alpha (TNF-α), B: interleukin-1β (IL-1 β) and C: interleukin-6 (IL-6) in the cerebral cortex of rats. All the data were analyzed using one-way ANOVA followed by LSD post Hoc test. Values are expressed as mean ± SD.; n = 6 rats for each group. a The mean values are significantly different compared to the control group at p< 0.05. b The mean values are significantly different compared to the tartrazin group at p< 0.05

For TNF-α: Variation between groups = 107.773, Variation within groups= .047 So, F = 2293.043

For IL-1 β: Variation between groups = 517.508, Variation within groups= 3.386 So, F = 152.838

For IL-6: Variation between groups = 90.251, Variation within groups= .031So, F = 2911.32

Fig. 4: Photomicrographs of sections in the cerebral cortex of rats. **A&B:** Sections in control rats , showing normal histological structure of the cerebral cortex layers; molecular layer (1), outer granular layer (II),outer pyramidal layer (III), inner granular layer (IV), inner pyramidal layer(V) and the multiform layer (VI). Note, normal granular cells (black arrows), blood vessel (red arrow) and normal neuropil (black arrow's head). **C&D**: Sections in melatonin-treated rat showing normal structure of the cerebral cortex with its 6 cellular layers ( I -VI). Note, normal pyramidal cells (black dotted arrow), granule cells (black arrow), neuroglial cells (green dotted arrow), blood vessel (red arrow) and normal neuropil (black arrow's head). **E&F**: Sections in tartrazin-treated rats, showing degenerated pyramidal neurons (black Square), irregular pyramidal cells with darkly stained nuclei (blue dotted arrows) , pericellular vacuoles (green arrow), vacuolated neuropil (red arrows head), pyknotic nuclei (yellow dotted arrows), red neuron (black arrows head), pericellular edema (green arrows), dilated and congested blood vessels (red arrows) and glail cells (black circle). G&H: Sections in, showing improvement in the granular cells (black arrows) and pyramidal cells (black dotted arrow). Some dilated blood vessels (red arrow), few neurons with pyknotic nuclei (yellow dotted arrow) and few pericellular vacuoles (green arrow) were observed. (H& E stain).

Fig. 5: Photomicrographs of sections in the cerebellar cortex of rats. **A&B:** Sections in control rats, showing the normal three layers; outer molecular (ML) with interneurons (green circle), purkinje layer (PL) with purkinje cells (black arrows) and inner granular cell layer (GL) consisting of small rounded granular cells. **C&D**: Sections in melatonin-treated rats showing normal structure of the previously mentioned three cellular layers, outer molecular, purkinje layer and inner granular cell layer. **E&F:** Sections in the cerebellar cortex from tartrazin-treated rat, showing molecular layer with prominent perineuronal vacuoles, irregular shaped Purkinje cells with darkly stained nuclei (black arrows) and remnants of degenerated Purkinje cells (black squares), whit matter (W) with vacuoles (green arrows), granular layer (GL) consisting of clumped granular cells with deeply stained nuclei (red circle), areas of degenerated cells and fibers (black square) and scattered interneuron cells (green circles). **G&H** sections from tartarazin+ melatonin-treated rat, showing improvement in the structure of molecular layer with interneuron cells (green circle), purkinje cells (black arrows) in purkinje layer (PL) and granular cells in granular layer (GL). Few vacuolated cells ( green arrows) were noticed.

Fig. 6: Photomicrographs of coronal sections in rats brain, showing immunoreactivity for Caspase-3, Bcl2, GFAP ( in cerebral cortex) and CYV( in cerebllar cortex). Sections in control and melatonin treated rats showed negative or faint positive reaction for both Caspase-3 and GFAP and a strong stain affinity for Bcl2 and CYV. On the other hand, brain sections from tartrazine-treated rats showed strong reaction for Caspase-3 and GFAP and weak reaction for Bcl2 and CYV. Cortex coronal sections in rats co-treated with melatonin+ tartrazine showed decreased number of both caspase-3 and GFAP positive cells and increased number of both Bcl2 and CYV positive neurons.
